# The immunological synapse: the gateway to the HIV reservoir

**DOI:** 10.1111/imr.12080

**Published:** 2013-06-16

**Authors:** Deanna A Kulpa, Jessica H Brehm, Rémi Fromentin, Anthony Cooper, Colleen Cooper, Jeffrey Ahlers, Nicolas Chomont, Rafick-Pierre Sékaly

**Affiliations:** 1 Division of Infectious Diseases, Vaccine and Gene Therapy Institute-Florida (VGTI-FL)Port Saint Lucie, FL, USA

**Keywords:** immunological synapse, virological synapse, HIV latency, HIV reservoir, asymmetric cell division, Wnt/Notch

## Abstract

A major challenge in the development of a cure for human immunodeficiency virus (HIV) has been the incomplete understanding of the basic mechanisms underlying HIV persistence during antiretroviral therapy. It is now realized that the establishment of a latently infected reservoir refractory to immune system recognition has thus far hindered eradication efforts. Recent investigation into the innate immune response has shed light on signaling pathways downstream of the immunological synapse critical for T-cell activation and establishment of T-cell memory. This has led to the understanding that the cell-to-cell contacts observed in an immunological synapse that involve the CD4^+^ T cell and antigen-presenting cell or T-cell–T-cell interactions enhance efficient viral spread and facilitate the induction and maintenance of latency in HIV-infected memory T cells. This review focuses on recent work characterizing the immunological synapse and the signaling pathways involved in T-cell activation and gene regulation in the context of HIV persistence.

This article is part of a series of reviews covering HIV Immunology appearing in Volume 254 of *Immunological Reviews*.

## Introduction

Lymph nodes (LNs) were identified as major sites of viral replication in HIV-infected subjects 1, 2. Both solid and diffused secondary lymphoid organs (SLOs), such as the gut-associated lymphoid tissues (GALT), were also shown to be the primary sites of viral replication in human immunodeficiency virus (HIV)/simian immunodeficiency virus (SIV) infections 3, 4. A large number of studies in humans and non-human primates have reported higher frequencies of HIV/SIV-infected cells as well as higher copy numbers of viral transcripts in CD4^+^ T cells isolated from lymphoid tissues (GALT, LN) when compared with the peripheral blood 1, 5–8. This enrichment in HIV-infected cells can be attributed to several important characteristics of lymphoid tissues. These include (i) a privileged tissue architecture that favors close cellular contact between immune cells, thereby promoting cell-to-cell transmission of HIV and ensuring viral dissemination; (ii) a significant enrichment in the frequency of cells that are highly permissive to HIV infection, such as activated CD4^+^ T cells that can produce large numbers of viral particles; and (iii) a proinflammatory environment that enhances viral production from infected cells and promotes new infections. These factors contribute to the high levels of HIV replication observed in lymphoid organs from HIV- infected subjects and provide an explanation of the major role played by these compartments in the pathophysiology of HIV infection.

**Fig. 1:**
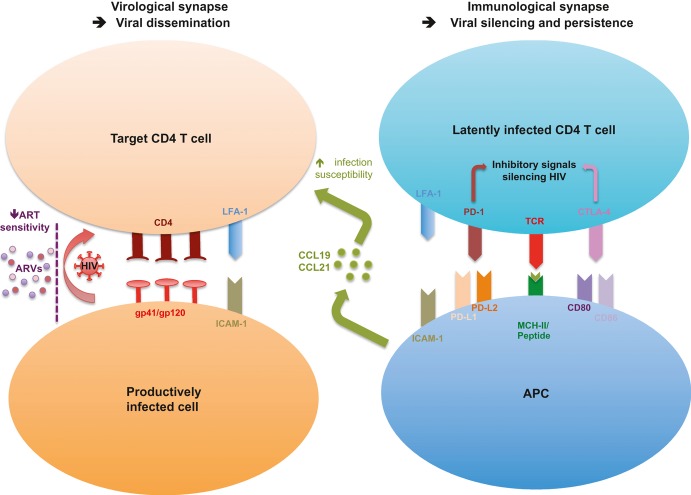
Model of virological and immunological synapse formation in the contribution to HIV persistence Virological synapse formation (left panel) is mediated through interactions of gp41/gp120 (shown in red) on an HIV-infected CD4^+^ T cell with CD4 (brown) on the cell surface of an uninfected target CD4^+^ T cell. Interactions are stabilized by ICAM-1 (green) and LFA-1 (blue) and take place even in presence of ART. Immunological synapse formation (right panel) initiated through interactions of MHC class II (green) on an antigen-presenting cell (APC) and the TCR (red) of an infected T cell may induce latency via inhibitory signals within the immunological synapse to reduce T-cell activation. These inhibitory signals include CTLA-4 (pink) on HIV-infected CD4^+^ T cells interacting with CD80/CD86 (purple/lavender) on APCs, or PD-1 (brown) with its ligand PD-L1 or PD-L2 (tan/orange). These interactions are localized to the pSMAC and are stabilized by LFA-1 and ICAM-1.

High frequencies of HIV-infected cells have also been reported in lymphoid tissues from subjects who have received suppressive antiretroviral therapy (ART) for prolonged periods of time 9–12, indicating that lymphoid organs are not only important sites of HIV production in untreated disease, but also play a major role in HIV persistence during therapy. The three aforementioned characteristics of lymphoid organs that contribute to HIV replication in untreated disease are likely to play a similar role during ART by promoting residual levels of viral replication 13. In addition to the close T-cell–T-cell and dendritic cell (DC)–T-cell contacts that favor viral transmission (the ‘virological synapses’), recent observations suggest that contact and crosstalk between immune cells (the ‘immunological synapse’) may play a critical role in HIV persistence by promoting the establishment and maintenance of viral latency 14. Therefore, although the immunological synapse and virological synapse share several common features, they may result in very distinct outcomes when their impact on viral persistence is examined (*Fig.* 1). Whereas the virological synapse functions to promote viral dissemination, the immunological synapse may result in the inhibition of viral production and in the establishment of HIV latency, thereby generating and maintaining a long-lived cellular reservoir for the virus. Details of the mechanisms by which the immunological synapse and virological synapse contribute to HIV persistence are still largely unknown. Our current understanding of these mechanisms and their downstream signaling pathways are detailed in this review. Identifying the cell types and receptors at play in these interactions will pave the way for the rational design of novel therapeutic approaches aimed at abrogating HIV persistence during ART.

## The HIV reservoir

Current antiretroviral regimens dramatically suppress HIV replication resulting in a major reduction in HIV-related mortality and morbidity; however, these treatments do not eradicate HIV. Interruption of ART almost invariably leads to the reemergence of detectable viral replication even after years of continuous optimal suppressive therapy, thereby demonstrating the presence of a long-lived viral reservoir constituted of a pool of cells capable of producing replication-competent HIV 15. Two non-mutually exclusive mechanisms underlie HIV persistence during ART: (i) incomplete suppression of viral replication could allow the continuous replenishment of a small pool of infected cells, particularly in sites with suboptimal drug penetration or sites in which control by the host immune system is inefficient and (ii) persistence of a small pool of resting memory CD4^+^ T cells in which HIV is maintained as a transcriptionally silent provirus through epigenetic mechanisms.

## HIV persists in a latent form through immunologically driven mechanisms

Fifteen years ago, several groups described a subset of resting memory CD4^+^ T cells with integrated HIV genomes that released infectious particles only upon cellular activation 7, 16. This latently infected reservoir was later shown to include mostly CD4^+^ T cells of central and transitional memory phenotype 17 and persisted in patients on ART who have no clinically detectable viremia 7, 18, 19. The kinetics of decay of the latently infected CD4^+^ T-cell subset has been shown to be approximately 60 years, as the mean half-life of CD4^+^ T cells was suggested to be 43.9 months 20–22. The establishment of a latent reservoir in these subsets is a rare event that occurs during acute infection and is not fully prevented by the introduction of ART 23, 24. Two major forms of viral latency coexist *in vivo* 25. First, preintegration latency refers to unintegrated HIV DNA that is unstable and will either degrade or integrate into the host cell genome, usually upon cellular activation 26–28. This form of latency is established after partial or complete block of the viral life cycle at steps prior to the integration of viral DNA. Second, postintegration latency refers to the presence of integrated HIV DNA in cells that are not actively producing viral particles. This latent state is extremely stable and is limited only by the lifespan of the infected cell and its progeny. Because the provirus is integrated in the host genome, its replication activity is significantly affected by the activation state of the host cell. Thus, CD4^+^ T cells may induce a latent state of the viral genome when transitioning from an activated effector phenotype to a quiescent memory cell.

Most mechanisms that lead to induction and maintenance of HIV latency operate at the transcriptional and epigenetic level. The first key factor is the site of provirus integration. Integrated HIV proviruses are typically found within actively transcribed genes in resting CD4^+^ T cells 29, 30. However, modifications such as histone acetylation, methylation, and adenosine triphosphate–dependent remodeling interfere with the transcription of viral genes by rendering the HIV LTR promoter region inaccessible to transcription factors 31–34. In addition, the quiescent state of latently infected CD4^+^ T cells favors nuclear exclusion of the transcription factors nuclear-factor κB (NFκB) and nuclear factor of activated T cells (NFAT) that promote HIV expression 35, 36. In quiescent CD4^+^ T cells, elongation factors such as pTEFB can also be sequestered and thus negatively impact the generation of viral transcripts 37, 38. Many of these mechanisms have been elucidated using cell lines and the relative importance of each mechanism *in vivo* remains unclear. However, the block in HIV production in quiescent memory CD4^+^ T cells extends beyond transcription, as low levels of cell-associated viral RNA have been found in resting CD4^+^ T cells from virally suppressed subjects 39. A defect in nuclear export of RNA transcripts has been suggested to block HIV production in latently infected cells 40.

A critical unanswered question pertains to the nature of signals an HIV-infected cell receives to establish and ultimately maintain a latently infected reservoir. The immunological mechanisms involved in the generation and maintenance of memory CD4^+^ T cells have been suggested to regulate the induction of latency and the persistence of the HIV reservoir 41. Several lines of evidence suggest that the generation of memory T cells from effector T cells during HIV infection contributes to the establishment of a reservoir of long-lived latently infected cells. Latently infected memory T cells harboring replication-competent HIV can be isolated from viremic donors 16, indicating that the latent HIV reservoir is generated and maintained during the viremic phase of the disease. Negative signals, notably mediated by negative regulators of T-cell receptor (TCR) signaling 42, may initiate the transition from activated to quiescent phenotype by reducing the availability of cellular transcription factors essential for active viral gene expression, thereby establishing viral latency in long-lived memory CD4^+^ T cells harboring HIV-integrated DNA. Memory CD4^+^ T cells persist in response to prosurvival signals downstream of common γ chain (γc) cytokines [such as interleukin-7 (IL-7) and IL-15] and TCR stimulation 43–45. We have demonstrated that these cytokines contribute to the persistence of HIV in this long-lived cellular compartment 17 by controlling homeostatic proliferation during ART 46, 47. Sequencing of HIV genomes in latently infected cells has revealed significant sequence homogeneity, which would support a model of homeostatic proliferation of a small number of latently infected cells 17. In contrast, a reservoir generated by ongoing viral replication and infection of new cells would be evidenced by an accumulation of mutations in the integrated HIV genomes 46, 47. Several immunological mechanisms could be responsible for proliferation-induced HIV persistence: (i) homeostatic proliferation driven by IL-7 and IL-15 48; (ii) inflammation-induced proliferation driven by proinflammatory cytokines such as IL-1, IL-6, and interferon-γ (IFN-γ) (49, discussed in this issue); (iii) antigen-induced proliferation; and (iv) self-renewal of stem cell memory T cells by Wnt/Notch signaling 50, 51. IL-7 or proinflammatory cytokines 52–54 as well as TCR engagement 55 have been shown to induce HIV production in primary CD4^+^ T cells *in vitro*. These results suggest that memory CD4^+^ T cells harboring replication-competent HIV may be continuously exposed to reactivation signals. The maintenance of viral latency may be an active process whereby the positive signals conferred by TCR stimulation and/or cytokines could be counterbalanced by negative signals that would impede viral reactivation and subsequent elimination of these cells through cytopathic effect or cytotoxic killing. Negative signals could also lead to asymmetric division and the establishment of long-lived stem cell memory T cells.

## Secondary lymphoid organs in HIV infection: exploring the battlefield

In addition to the persistence of HIV in a latent state within a small pool of long-lived CD4^+^ T cells, the incomplete suppression of viral replication by ART could allow the continuous replenishment of a small pool of infected cells 56, particularly in anatomical compartments in which drug penetration may not be optimal 10. Moreover, inflammatory cytokines such as IL-2, TNF (tumor necrosis factor), IL-6, and IL-18, and chemokines CC-chemokine ligand 19 (CCL19) and CCL21 are elevated during treated HIV disease; this provides the inflammatory environment that render CD4^+^ T cells more susceptible to infection in tissues of infected subjects 54, 57–59 (*Fig.* 1). Viral replication during ART is well documented by studies showing residual viremia in the majority of ART-treated subjects 60, 61, or the presence of cell-associated viral RNA in tissues such as the GALT 12. However, as discussed above the absence of viral evolution 62, 63 and the lack of impact of treatment intensification on residual viremia 64–66 argue against the role of viral production in HIV persistence. However, it cannot be excluded that specific microenvironments such as the immunological synapse could favor the continuous replenishment of a pool of HIV-infected cells. Indeed, evidence of cell-to-cell transmission of HIV in the presence of antiretroviral drugs has been recently described and indicates an important mechanism of HIV persistence 13 (*Fig.* 1).

SLOs provide an environment that enables lymphocytes to interact with antigen-presenting cells (APCs), resulting in the initiation of antigen-specific immune responses. SLOs are usually considered as sites of production of antigen-specific effector cells that have the ability to migrate to infected tissues and anatomical compartments in which infection by a pathogen takes place. This classical definition has to be revisited in the context of HIV infection, as the virus preferentially replicates in CD4^+^ T cells localized in SLOs. As a consequence, an effector response actively prevents viral dissemination in these SLOs when HIV-specific CTLs kill infected cells present locally 67. In the context of suboptimal adaptive immune responses characteristic of HIV infection, this duality is clearly beneficial to the virus by the continuous generation of new target cells through the interactions of APCs and CD4^+^ T cells via the immunological synapse. Of note, the innate immune response that includes natural killer (NK) cells, γδ T cells, NK T cells, and innate-like CD8^+^ T cells may also play an important role in limiting systemic pathogen spread in LNs 68, but whether these mechanisms may specifically impact HIV replication remains largely unknown.

Immunological synapse formation stimulates the differentiation of naive CD4^+^ T cells into effector helper cells through the recognition by the TCR of its cognate peptide–major histocompatibility complex class II (MHCII) complex on the surface of APCs 69. Two-photon imaging has revealed that naive T cells are in constant motion, scanning the lymph node in search of antigen by contacting 5000 DCs per hour 70, 71. The priming of naive T cells by a DC occurs in three distinct phases 72. During the first 8 h after entering the LN, T cells undergo multiple short encounters with DCs, decrease their motility, and upregulate activation markers such as the extended form of leukocyte function-associated antigen-1 (LFA-1) and cytotoxic T-lymphocyte antigen-4 (CTLA-4) (stop signal). To establish an immunological synapse, a stop signal results in the arrest of CD4^+^ T-cell migration to a particular DC 73, 74. During the subsequent 12 h, T cells form long-lasting conjugates with DCs and begin to secrete IL-2 and IFN-γ. On the second day, coinciding with the onset of proliferation, T cells resume their rapid migration and short DC contacts. The priming of antigen-specific T cells is highly sensitive, as the formation of an immunological synapse requires only about 10 agonist peptides presented at the surface of the APC 75.

Effector CD4^+^ T cells secrete cytokines that modulate adaptive immune responses, with IFN-γ and IL-2 promoting Th1 responses, whereas secretion of IL-4 and IL-5 promotes Th2 responses. Some of these cytokines are endowed with antiviral activity such as IFN-γ. APCs can also induce the generation of regulatory T cells (Treg), which can downmodulate antigen-specific immune responses 76. Interestingly, Treg cells can exhibit their regulatory function at the immunological synapse by preventing the recruitment of signaling molecules on naive T cells such as PKCθ when both T cells have identical antigen specificity and are in contact with the same APC 77. It has become more and more evident that the interactions occurring at the T-cell–APC interface determine the nature of the T-cell response against a particular antigen. We now examine our current understanding of the structure and signaling pathways of the immunological synapse.

## Spatial organization of the immunological synapse

The immunological synapse was originally characterized at the surface of T cells as concentric rings of membrane receptors 78. These three-dimensional contact domains, which are visible by confocal microscopy, are named supramolecular activation clusters (SMACs) and include the central SMAC (cSMAC), the peripheral SMAC (pSMAC), and the distal SMAC (dSMAC). During T-cell activation, TCRs accumulate into the cSMAC, surrounded in a bulls-eye manner by the pSMAC, which consists of a ring enriched in the adhesion molecule LFA-1 and its ligands, which are members of the intercellular adhesion molecule (ICAM) family and talin. Talin has been demonstrated to link the adhesion rings to the actin cytoskeleton 79. LFA-1 and ICAM-1 interactions act as a tether between the two cells, facilitating contact between the TCR and MHC and impeding T-cell mobility. Importantly, the stability provided by the pSMAC accounts for a 100-fold increase in the T-cell sensitivity to antigen 80. This organization (pSMAC and cSMAC) constitutes the mature immunological synapse. Finally, the most external ring or dSMAC is where proteins with large ectodomains such as CD43 and CD45 are located, far from the cSMAC 81. Importantly, the immunological synapse is dynamic: TCR signaling is sustained by TCR microclusters, made up of the TCR and peptide MHC complex that are continually forming in the dSMAC and moving into the cSMAC 82, 83. These newly formed microclusters are associated with signaling molecules such as ZAP-70 (ζ chain-associated protein kinase of 70 kDa), Lck (lymphocyte-specific protein tyrosine kinase), LAT (linker of activated T cells), and SLP-76 (SH2 domain–containing leukocyte protein of 76 kDa), but these associations are lost as the microclusters migrate toward the cSMAC. Therefore, TCR signaling is initiated and sustained in peripheral microclusters, and despite the large numbers of TCRs present in the cSMAC, signaling does not occur in the central part of the immunological synapse. Although the cSMAC may play several roles during the formation and termination of the immunological synapse, it may primarily serve to downregulate the TCR by endocytosis 84. The dSMAC also has CD45 and dynamic filamentous actin 85–87 and has been implicated in T-cell sensitivity to antigen recognition 73, 88–90. Significant for HIV infection, CD4 is initially recruited to the TCR–peptide–MHC microcluster in the cSMAC as well as the coreceptors CXCR4 and CCR5 91, 92.

## The immunological synapse: fine tuning of the immune response

The highly stable and long-lived immunological synapse has been demonstrated to be required to completely activate T cells following TCR engagement and its triggering of a signaling cascade 93, 94. In addition to the TCR, a large number of proteins play essential roles in the formation of immunological synapse. They include coreceptors, adhesion molecules, and costimulatory and negative regulatory molecules. CD4, through its extracellular domain, acts as a coreceptor to the TCR and interacts directly with MHCII molecules on the surface of APCs. Although CD4^+^ T cells can respond with transient calcium signaling to a single agonist peptide–MHC ligand, a complete immunological synapse and productive calcium response require about 10 peptide–MHC complexes 75. This sensitivity is highly dependent on CD4, as anti-CD4 antibodies render T cells unable to detect less than about 30 ligands. Using its intracellular domain, CD4 amplifies the signal generated by the TCR by recruiting Lck 95. Lck phosphorylates the intracellular chains of the CD3 and ζ-chains of the TCR complex, allowing the recruitment of ZAP-70. Lck also phosphorylates and activates ZAP-70, which in turn phosphorylates LAT, a transmembrane protein that recruits a number of proteins essential for proper T-cell activation, including GRB2 (growth factor receptor-bound protein 2) and PLC-γ1 (phospholipase C γ1).

Adhesion receptors can be defined as receptors that promote adhesion beyond antigen recognition 96. The arrest of rolling leukocytes on the endothelium is nearly exclusively mediated by members of the integrin superfamily, such as the myeloid-specific integrin LFA-1, Mac-1, as well as the two α4 integrins, α4β1 (VLA-4) and α4β7 97. Soluble factors such as chemokines also have been implicated in T-cell arrest. For example, CCL19 and CCL21 are expressed by the vasculature during inflammation and signal through CCR7 on leukocytes, which results in the unfolding of LFA-1 into an extended conformation, enabling it to bind to ICAM-1 with intermediate affinity 98, 99. In addition, T cells polarize in response to CCL19 and become highly motile 100. As a result, the efficiency of naive T cells in scanning the surface of APCs is improved, increasing the probability of encountering a cognate peptide–MHC complex 69. Chemokines such as CCL19 and CCL21 were recently shown *ex vivo* to increase susceptibility of resting memory T cells to infection and establishment of latency 58, 101.

## Regulatory molecules of the immunological synapse

Costimulatory and negative regulatory molecules can be defined as having a positive or a negative role in the regulation of TCR-mediated signals. Although some of these molecules may also have limited function outside the context of antigen recognition, costimulatory molecules play a critical role in the initiation of T-cell activation following the formation of the immunological synapse. For example, association of the TCR of a naive T cell with a peptide–MHC complex without interaction of the costimulatory receptor CD28 with its primary ligand CD80 (B7.1) results in an anergic T cell that produces very low amounts of IL-2 102. CD28 is highly enriched in TCR microclusters when engaged by CD80, and these CD28–CD80 complexes are transported to the center of the immunological synapse where they form a stable ring around the cSMAC 103. CD28 has a highly conserved short cytoplasmic tail that has no intrinsic enzymatic activity. However, phosphorylation of the tyrosine residues provides docking sites for SH2 domain–containing proteins, whereas the proline-rich motifs can bind SH3 domain–containing proteins. The role of CD28 costimulation on IL-2 production appears to have two stages: an initial phosphoinositol 3-kinase (PI3K)-dependent initial phase that acts on IL-2 transcription and a second phase which results in enhancement of IL-2 mRNA stability 104.

CD2 is also required for T-cell activation and cooperates with CD28 upon ligation to LFA-3/CD58 to induce the immunological synapse formation 105. CD2 binding with CD58 expressed on the surface of APCs augments and sustains antigen-induced Ca^2+^ increase in T cells 106. CD2 contributes to the generation of TCR triggered microdomains in the membrane that recruit signaling molecules like Lck and LAT 107 and play a direct role in T-cell signaling via multiple polyproline motifs that activate kinases such as Fyn. Like CD2, CD45 has been shown to be crucial for supporting signal transduction from the TCR. Through its intracellular region, CD45 associates with several intracellular protein tyrosine kinases essential for T-cell activation, including Lck, Fyn, and ZAP-70 108, 109. CD45 dephosphorylates Y505 on p56^lck^ activating Lck kinase, followed by p56^lck^ phosphorylation of TCR-ζ, and consequently recruitment of ZAP-70 108, 110. Interestingly, CD45 has been shown to negatively regulate the transcription factor NFAT, thereby reducing HIV LTR activation 111. Other costimulatory receptor–ligand pairs, including inducible costimulator (ICOS)–ICOS ligand (ICOS-L) 112, CD40–CD40L 113, CD6–CD166 114, and CD26–adenosine deaminase–adenosine receptor 115, were also reported to accumulate and function at the immunological synapse.

The formation of the immunological synapse and the subsequent T-cell activation can be inhibited by engagement of inhibitory receptors. The classic example is given by CTLA-4 (CD152), a negative regulator of T-cell activation that potently inhibits signaling through the TCR and reduces T-cell activation. HIV-specific CD4^+^ T cells upregulate CTLA-4 expression upon recognition of cognate peptide–MHC ligands leading to inhibition of effector function; blockade of this interaction augments HIV-specific CD4^+^ T-cell functions 116. Interestingly, CTLA-4 blockade augments viral replication in SIV-infected macaques 117, suggesting that this negative regulator may inhibit viral production *in vivo*, particularly in mucosal tissues where it is expressed at high levels (*Fig.* 1). This is consistent with data from our group that HIV Nef downregulates expression of CTLA-4, thereby maintaining CD4^+^ T-cell activation and viral replication 118. While T cells constitutively express CD28, CTLA-4 expression is induced by TCR stimulation 119. CTLA-4 has a much higher affinity for CD80 and CD86 than CD28. As a consequence, even low levels of CTLA-4 on the cell surface can compete for ligand binding with CD28. CTLA-4 forms microclusters, which directly accumulate in the cSMAC, exactly in the same region as CD28 120. Thus, CTLA-4 pushes CD28 away from the cSMAC, which results in the blockade of CD28-mediated costimulation 121. This is thought to be the main mechanism of CTLA-4–mediated inhibition of T-cell activation. CTLA-4 reverses the TCR-mediated stop signal needed for T-cell/APC interactions, thereby reducing the contact time between T cell and APC. This greatly reduces the contact area at the immunological synapse that leads to a major reduction in Ca^2+^ mobilization and IL-2 production 122, 123 and could also lead to significant inhibition of HIV replication. This signaling cascade results in cytoskeletal changes and microtubule-organizing center polarization toward the APC at the immunological synapse 124–126, resulting in the redistribution of the secretory and recycling machineries of the target T cell and a polarized transport of cytokines and signaling molecules toward the immunological synapse in the absence of cell fusion 80, 127.

PD-1 (programmed death-1) is a relatively new member of the extended B7-CD28 family of T-cell regulatory molecules. PD-1 is not detectable on naive T cells, but its expression is induced in T cells, B cells, and myeloid cells after activation 128. PD-1 expression is also upregulated in T cells upon exposure to γc cytokines IL-2, IL-7, IL-15, and IL-21 129. The expression of PD-1 is particularly high on the surface of functionally exhausted T cells in multiple persistent viral infections in humans 130, 131, particularly during chronic HIV infection 132–134, and is associated with various measures of viral persistence (ultrasensitive plasma HIV RNA levels, cell-associated HIV RNA levels, and proviral HIV DNA levels) 135. In addition, PD-1^+^ CD4^+^ T cells constitute a preferential reservoir for HIV 17. The interaction between PD-1 and its ligand PDL-1 has been shown to suppress HIV production in primary CD4^+^ T cells from viremic and virally suppressed subjects 136.

Signaling pathways initiated upon the interaction of PD-1 with its ligands (PDL-1/PDL-2) negatively regulate signals downstream of the TCR 137 and dampen cytokine production and proliferation 138. The level of PD-1 expression positively correlates with the degree of functional exhaustion, but this phenotype is actively maintained by signaling. PD-1 inhibits T-cell activation by pathways distinct from CTLA-4 139. PD-1 engagement leads to the inhibition of Akt phosphorylation by preventing CD28-mediated activation of PI3K. Using single-cell imaging, a recent study elucidated a molecular mechanism of PD-1–mediated suppression 140. Upon binding to PDL-1, PD-1 becomes clustered with TCRs and is transiently associated with the phosphatase SHP2 (Src homology 2 domain–containing tyrosine phosphatase 2). These negative costimulatory microclusters induce the dephosphorylation of the proximal TCR signaling molecules, resulting in the suppression of T-cell activation. PD-1 ligation is more effective than CTLA-4 in suppressing CD3/CD28-induced changes in the T-cell transcriptional profile, suggesting that differential regulation of PI3K activation by PD-1 and CTLA-4 ligation results in distinct downstream cellular outcomes 139. Other negative regulators such as LAG-3 (lymphocyte-activation gene 3) 141 and BTLA (B- and T-lymphocyte attenuator) 142, which have been shown to localize at the immunological synapse, may exert a similar role. The engagement of negative regulators of T-cell activation such as PD-1, CTLA-4, and others induces epigenetic changes 143 and may regulate histone deacetylase (HDAC) activity induced by CD3/CD28 T-cell activation. These observations suggest a key role for multiple negative regulator molecules in the establishment and maintenance of the latent HIV reservoir (*Fig.* 1).

## Molecular interactions that govern virological synapse formation

Along with co-opting the immunological synapse, another mechanism HIV employs to enhance viral spread is the direct transfer of virus between infected and uninfected CD4^+^ T cells (*Fig.* 1). Virological synapse formation is initiated via the interaction between surface HIV gp120 on the infected donor T cell and CD4 molecules on the surface of the uninfected target cell, in the absence of TCR–peptide–MHC interaction. Compared with the immunological synapse, the virological synapse is short lived, with a mean duration of 60 min 144 and completion of virus transfer within 3 h 145, 146. Gp120 and CD4 molecules are at the center of the microcluster and have been proposed to form a cSMAC-like structure similar to that observed for the TCR:MHC microclusters in immunological synapse formation 147–149. Like the immunological synapse, LFA-1 and ICAM-1 have been shown to assemble into an adhesive ring in a pSMAC-like structure in the virological synapse 83, 150. Unlike the immunological synapse, however, other molecules are required for virological synapse formation; their localization and their role in HIV cell-to-cell transmission are not as clearly defined and are still the subject of investigation. The first descriptions of the virological synapse by the Sattentau group 146 indicated the involvement of HIV Env and Gag from the HIV-infected donor cell, and CD4, LFA-1, and CXCR4 enrichment in the target cell at the point of contact. Studies using inhibitors of CD4 and Env have confirmed the requirement for these molecules in cell-to-cell HIV transmission, but HIV coreceptor antagonists have failed to inhibit the process 145, 151. Additional data using antibodies which block gp120–CD4 binding suggest the complex between gp120 and CD4 initiates virological synapse formation without requiring either CXCR4 or CCR5 coreceptor molecules 145, 149; however, HIV coreceptors may be required for later steps, after synapse formation, in the target cell 152. Recent studies as to the role of Gag in virological synapse formation have determined that the matrix (MA) domain is required for cell-to-cell HIV transmission and that Gag is specifically and directionally recruited into a disk-shaped structure called a synaptic button 145, 153, 154. Accumulation of Gag in button structures is indicative of new particle assembly. However, Gag has also been reported to aggregate into ring-like structures 155. The significance of the different structures Gag can form during virological synapse formation is not yet completely understood.

Other cellular factors implicated in HIV virological synapse include the LFA-1 ligands ICAM-2 and ICAM-3, tetraspanins, lipid raft marker GM-1, and integrin α4β7 156–159. For cell-free routes of HIV infection, LFA-1 binding to ICAM-1 has been demonstrated to enhance viral particle infectivity and decrease the effects of neutralizing antibodies by involvement in virus fusion 160. The role of the interaction of LFA-1 and its ICAM ligands during virological synapse formation remains unclear. It has been hypothesized that this complex may play a role in virological synapse formation similar to that observed in the immunological synapse that of pausing CD4^+^ T-cell migration to allow for the interaction of the target and donor cells 152. A study by Vasiliver-Shamis *et al*. 148 demonstrated that CD4^+^ T-cell interaction with gp120 and ICAM-1 in a virological synapse results in a TCR signaling cascade similar to that observed in an immunological synapse, such as the phosphorylation of Lck, CD3ζ, ZAP70, LAT, SLP-76 Itk, and PLCγ. In contrast, they did not observe recruitment of PKCθ or intracellular calcium mobilization, which may indicate that activation of the target CD4^+^ T cell through the virological synapse is incomplete 148. This is significant for HIV infection, as CD4^+^ T cells that are quiescent (G_0_) are highly resistant to infection; however, incomplete activation that results in progression to the G_1b_ phase results in increased susceptibility to HIV infection 28, 161. Consequently, suboptimal stimulation of the target CD4^+^ T cell through the virological synapse may be all that is required for productive infection of an otherwise resistant T cell. This would be conducive to HIV infection of long-lived memory CD4^+^ T cells and may be one mechanism leading to the establishment of the HIV reservoir in HIV-infected subjects.

Another requirement for HIV cell-to-cell transmission is the presence of lipid raft domains and cholesterol in the virological synapse found by Jolly and Sattentau 157. During viral assembly in infected cells, HIV is focused in GM-1-rich lipid rafts at the plasma membrane 162–169. In the virological synapse, lipid rafts in the infected donor cell are polarized to the site of the cell–cell contact 157. The role of lipid rafts in virological synapse formation in the recruitment of downstream signaling molecules or the stabilization of cell–cell contact remains to be determined.

Actin remodeling has also been shown to be required for virological synapse formation. Actin has been observed to accumulate at the synapse site in the infected donor CD4^+^ T cells 146, 170. Actin polymerization is induced by gp120 binding with CD4 and is required for CD4 and coreceptor recruitment to the virological synapse 171–173. In the target CD4^+^ T cell, an opening in the actin structure has been observed 148. This opening has been hypothesized to be critical for successful cell-to-cell transmission, as polymerized actin can act as a physical barrier that reduces viral infectivity post entry 174, 175. The precise roles for the actin cytoskeleton in both donor and target cell in the virological synapse and the recruitment of necessary factors are still under investigation.

## The virological synapse: a vehicle for HIV persistence

The potential significance of cell-to-cell HIV transmission is clear, as studies have demonstrated that cell-associated HIV can have 100–1000 times the infection efficiency of cell-free virus 144, 145, 176–178. Given this efficiency, it may be hypothesized that a cell-to-cell infection route may result in the transfer of more than one virion per synapse. Indeed, several studies have indicated the generation of a high local MOI 179, 180, which could be visualized in time-lapse microscopy 154, 181, 182. This would arise from the presence of multiple virions potentially present at the site of transfer that may enter the target cell before mechanisms to suppress multiple infection, such as downregulation of CD4, can take place. Support for this mechanism *in vivo* comes from the observed higher HIV DNA copy number per infected cell derived from tissues, whereas peripheral blood CD4 may only have a single provirus per infected cell 183.

A new study performed in humanized BLT (bone marrow/liver/thymus) mice has offered additional insights into the significance of cell-to-cell transmission *in vivo* and provides an intriguing model in which to study this phenomenon. One of the advantages offered by humanized BLT mice is the fact that the system also allows both naive and memory CD4^+^ T-cell homing to SLOs, as indicated earlier are localized sites of high numbers of CD4^+^ T cells as potential target cells for viral replication and transmission. Indeed, the study by Murooka *et al*. 184 did find evidence for tethering interactions that indicated virological synapse formation. However, their study also reported approximately 10–20% of infected cells in the lymph node to be multinucleated syncytia 184. For most CD4^+^ T-cell models of virological synapse formation, syncytia are not normally observed 185. The short duration of the virological synapse compared with the immunological synapse may be a mechanism to reduce cell fusion events during cell-to-cell transmission of HIV. Another mechanism is the recruitment of cellular factors to the virological synapse that actively inhibits cell–cell fusion such as regulatory tetraspanins. Tetraspanins are a class of surface molecules that have a variety of functions in the regulation of cell signaling and adhesion. Tetraspanins such as CD9, CD63, and CD81 have been shown to inhibit cell–cell fusion 186. Several studies have demonstrated a requirement for CD63 and CD81 in the virological synapse specifically in cell-to-cell transmission 186–188. High expression levels of tetraspanins were also demonstrated to reduce syncytium formation in a gag-dependent manner 187.

Whether syncytia as a common outcome of cell-to-cell HIV transmission *in vivo* are accurately recapitulated in the BLT mouse model or are a product of the experimental model background remains to be determined. Moreover, Murooka *et al*. 184 report a difference between the results of HIV infection in the BLT model system and another *in vivo* model system for studying HIV infection, SIV infection of Rhesus macaques. In the macaque model system, most SIV-infected CD4^+^ T cells are resting memory phenotype 189, whereas in the BLT mouse model the majority of the infected CD4^+^ T cells in the lymph node were resting, CD45RO^+^ cells of central or effector memory phenotype 184. In determining the role of cell-to-cell HIV transmission between CD4^+^ T cells *in vivo*, the phenotype of both the donor and target CD4^+^ T cell may provide important clues as to how HIV infection is initiated and ultimately how a latently infected reservoir is established. For example, it is well known that CD4^+^ immune cells at different activation states have varying rates of permissiveness for HIV infection, with the more activated CD4^+^ effector memory T-cell subset being the most permissive 27. If HIV infection was maintained solely in activated effector T cells, then the immune system may be more able to eradicate the virus due to a greater likelihood of ongoing viral replication in these cell types and recognition by effector T cells. However, it has been demonstrated previously that HIV is present in both central and effector memory CD4 subsets 17, and the mechanism of infection of these different subsets and establishment of the latent HIV reservoir in each is still poorly understood. The virological synapse may play a more significant role in establishment of the latent reservoir than previously thought, if it can be demonstrated *in vivo* that the interactions between CD4^+^ T cells themselves or between CD4^+^ T cells and APCs in the lymph nodes enhanced infection rates resting CD4^+^ T cells that have been shown to be more resistant to infection 26, 28, 190. If these CD4^+^ target cells that are more naturally resistant due to a lower activation state or quiescence become transiently, suboptimally activated during formation of the virological synapse, the target cell then may rapidly return to a quiescent state and be maintained as a long-lived memory cell carrying proviral DNA. What is more, the mechanism of cell-to-cell transmission may also allow HIV to spread without triggering immune system detection, as several studies have suggested that virological synapse transmission of virus limits the epitope exposure of HIV gp120 to broadly neutralizing antibodies 191, 192. Altogether, these data suggest a new paradigm for establishment of HIV infection that encompasses virological synapse formation and cell-to-cell HIV transmission as a key mediator of HIV persistence and defines new challenges on the road to eradication.

## The role of the immune synapse in HIV latent reservoir maintenance

The immunological synapse represents a crucial point of communication between T cells and APCs through which the APC can direct the T cell to a number of different cell fates. Significantly, this interaction may contribute to the maintenance of the latent HIV reservoir through various mechanisms. For example, the context of the immune synapse can influence the effector function of HIV-specific T cells resulting in diminished anti-HIV responses. Likewise, the immune synapse may be able to alter the biology of HIV-infected cells rendering the HIV silent for long periods of time. This section seeks to identify ways in which this critical component of the immune response may be contributing to the maintenance of the latent HIV reservoir.

## Immune synapse and induction of stemness as a mechanism of latency

As detailed above, one mechanism of HIV persistence during prolonged ART is the long-term survival of latently infected quiescent memory CD4^+^ T cells, which may serve as a reservoir that contributes to viral load rebound after cessation of ART. This mechanism can potentially be explained with the stem cell–like nature of memory T cells, which has become a point of recent interest 193. Memory CD4^+^ T cells share several characteristics with stem cells, most notably, they are long-lived, capable of self-renewal, and able to differentiate upon stimulation; latently HIV-infected memory CD4^+^ T cells share these traits. Indeed, a population of memory T cells was recently identified with stem cell–like properties 194. Using a mouse model, stem cell memory T cells (Tscm) were found to display a gene signature intermediate of naive T cells and central memory T cells (Tcm). Tscm are CD45RA^+^CD95^+^IL-2Rβ^+^ and demonstrated the robust reconstitution potential of memory T cells. In non-human primate models, this same population was observed during the acute phase of viral infection and was maintained long term, compared with other memory subsets, after the removal of antigen 195. Although HIV infection in this population has not been demonstrated, it stands to reason that a ‘reservoir of T-cell memory’ could also be a reservoir of HIV latency.

While little is yet known about how the mechanisms that lead to differentiation into stem cells T cells can be induced to become T-cell stem cells, observations of embryonic stem cells and inducible pluripotent stems can give some prediction as to what signals could be promoted by the immunological synapse. Negative regulators of T-cell signaling may be connected to signals that are known to provide critical stem cell functions. Indeed, several transcriptional coactivators have been described that change the outcome of TCR stimulation when present in the context of an immune synapse. One such example is the Yes-associated protein (Yap), a protein required for self-renewal of embryonic stem (ES) cells, which is responsible for a transcriptional profile of pluripotency genes that include Oct4, Nanog, and Sox2 196. Yap expression has recently been implicated in self-renewal and memory precursor maintenance in CD8^+^ T cells and is inhibited by activation of Akt and the Hippo pathway 197. Activation of PD-1 on T cells inhibits the activation of Akt, which may allow for sustained Yap expression and direct a more stem-like quality in the T cell 139. This observation suggests a mechanism by which the composition of molecules at the immune synapse may be able to shift an infected T cell toward a long-lived, stem-like memory cell. Yap expression in the context of Wnt signaling is another mechanism associated with stem cell self-renewal and regulation of differentiation. In colorectal cancers, the canonical Wnt signaling complex, β-catenin/TCF4 (T-cell factor 4), drives Yap expression 198. More recently, it was proposed that after Yap is phosphorylated by LATS1/2 (large tumor suppressor 1/2) kinases, it is retained in the cytoplasm where it interacts indirectly with disheveled (Dvl) and inhibits nuclear translocation of Dvl, blocking TCF transcription of Wnt target genes 199.

Another potential promoter of stemness in HIV-infected CD4^+^ T cells comes from cytokines secreted at the site of the immune synapse. One example is signal transducer and activator of transcription-3 (STAT-3), a transcription factor that links cytokine signals to pluripotent stem cell potential. Activation of STAT-3 is required for self-renewal of ES cells 200, 201, and its critical role in T cells is supported by the identification of patients with a mutation in STAT-3 that have dysfunction in the maintenance of T-cell memory 202. Furthermore, recent observations in CD4^+^ T cells point to STAT-3 activation downstream of IL-21 as a mechanism for maintaining stem cell–like qualities in Th17 cells 202. In addition to the importance of STAT-3 signaling in stem cell potential, these experiments also point to a role for β-catenin Wnt signaling and Notch signaling.

## Wnt signaling pathways and gene regulation

Wnt signaling is a fundamental component in embryonic stem cell proliferation, differentiation, and cell fate decisions 203–206, homeostasis in adult stem cells and tissues, and self-renewal in cancer stem cells (reviewed in 207). The diversity of receptors, ligands, and regulators 208 that comprise the Wnt signaling pathway provide the foundation to influence cell development and homeostasis in a variety of organs/tissues.

Wnt signaling is triggered through either a β-catenin–dependent (canonical) or -independent (non-canonical) pathway by interactions between one of 19 Wnt ligands with one of 10 seven-transmembrane receptors termed Frizzled (Fzd). Non-canonical signaling does not rely on gene expression associated with β-catenin. Signaling occurs when Wnt ligands specific to non-canonical signaling binding to Fzd receptors and coreceptors other than Lrp4 [Ldl (low density lipoprotein) receptor-related protein 4], Lrp5, Lrp6, or no coreceptor 209. The most well-characterized non-canonical pathways are the planar cell polarity pathway and the Ca^2+^-dependent pathway (reviewed in 210, 211).

**Fig. 2:**
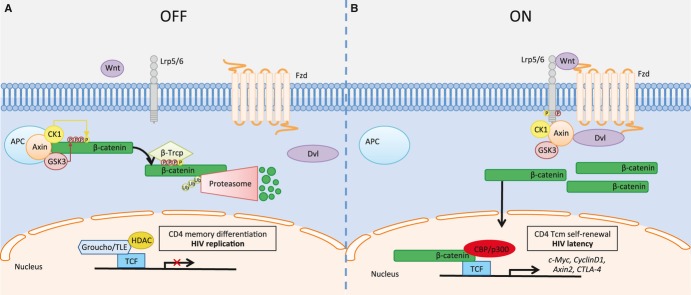
Canonical Wnt signaling and its potential role in T-cell differentiation, self-renewal, and HIV latency (A) In the absence of Wnt, the destruction complex composed of APC, Axin, Ck1, and GSK3-β binds to and phosphorylates cytoplasmic β-catenin. Phosphorylated β-catenin is ubiquitinated by β-TrCP followed by proteasomal degradation. In the current model of canonical Wnt signaling, β-TrCP ubiquitination and proteasome degradation occurs as a separate complex from the destruction complex, whereas β-TrCP and the proteasome are part of the destruction complex in a new model proposed by Li *et al*. 214. Within the nucleus, TCF/LEF and the help of transcriptional corepressors (Groucho) and HDACs repress target genes. ‘Wnt-off’ favors CD4 memory T-cell differentiation and may influence HIV replication. (B) In the presence of Wnt ligands, Wnt binds to Fzd receptor and Lrp5/6 coreceptor to initiate signaling. CK1γ and GSK3β phosphorylate Lrp5/6 at the plasma membrane and the interaction between CK1γ, GSK3β, Axin, and Dvl inactivates/blocks ubiquitination of phosphorylated β-catenin [new model 214] or causes a dissociation of the destruction complex (current model). Free, unphosphorylated β-catenin enters the nucleus and displaces corepressors from TCF/LEF and recruits transcriptional coactivators and histone modifiers such as CBP/p300, Pygo, and Bcl9 to drive target gene expression. ‘Wnt-on’ favors Tcm self-renewal and induction of HIV latency.

β-catenin is the key component to canonical Wnt signaling and mediates both structural and signaling functions. In the absence of Wnt signaling, a destruction complex comprised of glycogen synthase kinase 3β (GSK3β), casein kinase 1 (CK1), axin and adenomatous polyposis coli (APC) phosphorylate β-catenin, and target the phosphorylated form for unbiquitin-dependent degradation by the E3 ligase β-transducin repeat-containing protein (β-TrCP) (*Fig.* 2). In the presence of Wnt ligands, Wnt binds to its receptor, Fzd, and coreceptor, Lrp, to induce Lrp phosphorylation mediated by GSK3β and CK1. Through an unclear disheveled (Dvl)-dependent mechanism, unphosphorylated β-catenin accumulates and translocates into the nucleus where β-catenin replaces Groucho and corepressors from DNA-bound T-cell factor (TCF)/lymphoid enhancer factor (LEF) and recruits transcriptional coactivators and histone modifiers to drive expression of genes that promote cell cycle and survival (cyclinD1, c-Jun, fra-1, c-myc). CTLA-4, a negative regulator of T-cell activation and correlate of HIV latency, is upregulated by Wnt3a in human melanoma tumors 212 and differentially expressed along with the negative regulator PD-1 in activated CD4^+^ T cells (J.H. Brehm and R.-P. Sekaly, unpublished data). A comprehensive list of Wnt target genes can be found at http://www.stanford.edu/group/nusselab/cgi-bin/wnt/target_genes 213, 214.

The structural function of β-catenin is defined by interaction with cadherins at the plasma membrane, mediating an indirect role in cell-to-cell adhesion and reduced proliferation. Increased expression of cadherins in human SW480 human colon carcinoma cells recruits β-catenin to adherin junctions at the cell surface, decreasing nuclear pools of β-catenin, and thereby antagonizing β-catenin–LEF/TCF transcription of genes associated with proliferation 215, 216.

Wnt signaling transcription factors TCF1 and TCF3 have activator and repressor functions, respectively, in embryonic stem cell self-renewal and determination of stem cell fate. In the absence of the mitogen-activated protein kinase (MAPK) extracellular signal-regulated kinase (ERK) and GSK3β inhibition, TCF3 bound to DNA represses genes specific to self-renewal (Oct4, Sox2, Nanog, and Klf2/4) and drives stem cell differentiation. Conversely, in the presence of MAPK (ERK) kinase and GSK3β (Wnt signaling) inhibition, genes that maintain stem cell self-renewal are expressed after β-catenin removal of TCF3 repression followed by transcriptional activation of TCF1 and stimulation of biosynthetic and metabolic processes 204, 205. Genes regulating Wnt signaling activity are often differentially regulated in T-cell populations, as seen from microarray data analyzed at VGTI-FL. It will be interesting to interpret these data and what their expression means related to Wnt signaling activity specific to self-renewal and survival of memory T cells.

## Role of Wnt/β-catenin signaling in stem cell memory T cells and HIV latency

Components of both the canonical and non-canonical Wnt signaling pathways are active in T lymphocytes. For instance, CD8^+^ naive T cells (Tn) and Tscm highly express Wnt signaling transducers, TCF7 and LEF1, that are lost during differentiation from Tn to Tscm to Tcm to effector memory T cells (Tem) 194. In fact, Wnt3a ligand and inhibitors of GSK3β inhibit the differentiation of Tn to short-lived effector T cells (Teff) while enhancing self-renewal of CD4^+^ and CD8^+^ Tcm cells 50, 51 and CD8^+^ Tscm 50 (*Fig.* 2). Furthermore, gain-of-function and loss-of-function studies of β-catenin and TCF1 in CD8^+^ T cells from mice confirm that canonical Wnt signaling is sufficient for the establishment of long-term Tcm cells 217–219.

Consistent with these findings, preliminary observations in our laboratory indicate that memory CD4^+^ T cells express Wnt receptors (FZD1-10 and LRP6). After stimulation of memory CD4^+^ cells with anti-CD3/CD28 in the presence of Dickkopf-related protein 1 (Dkk1), an inhibitor for canonical Wnt/β-catenin signaling, the number of proliferating cells increased and phosphorylation of β-catenin (ser33/37/Thr41) decreased (C. Benne and R.-P. Sekaly, unpublished data), implying that Wnt/β-catenin signaling may maintain self-renewal and survival of memory CD4^+^ T cells. In fact, diminished ki67 and CD38 expression on memory CD4^+^ T cells and preservation of the Tcm subset were observed after a 7-day culture in the presence of Wnt3a and anti-CD3/CD28 (J.H. Brehm and R.-P. Sekaly, unpublished data). These findings in CD4^+^ T cells in combination with published results strongly suggest the canonical Wnt/β-catenin pathway as a critical component in self-renewal of CD4^+^ Tscm and Tcm populations and may be a mechanism for maintaining cells containing the HIV latent reservoir.

## Notch signaling and gene regulation

Similar to Wnt, Notch signaling is a key factor in mediating stem cell proliferation, self-renewal, differentiation, and quiescence in various tissues associated with embryonic development and adult tissue homeostasis. Notch signaling is context dependent; thus, the microenvironment of cells in which Notch is signaling will strongly influence their fate. Notch signaling in stem cells and other tissues has been elegantly reviewed by Koch *et al*. 220. In context of the immunological synapse, combined Notch signaling and TLR stimulation of DCs can modulate TLR-induced cytokine expression compared with TLR or Notch signaling alone, including increased IL-2 and IL-10 expression and decreased IL-12 expression. This mechanism is dependent on PI3K activity after signaling through an alternate Notch pathway than the canonical Notch pathway described below 221. DC–T-cell interactions and the resulting immune response may be affected in an environment influenced by Notch/TLR ligand signaling.

**Fig. 3:**
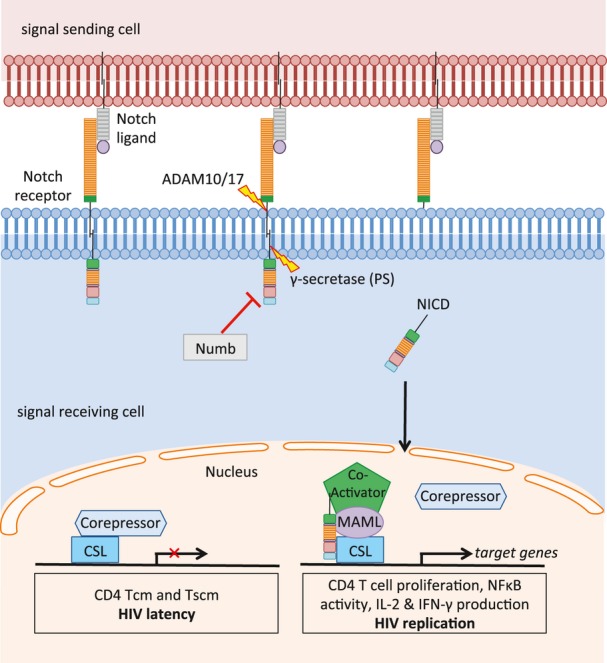
Role of the canonical Notch signaling pathway in asymmetric cell division and HIV latency Notch ligand on a signal-sending cell binds [Jagged1, Jagged2, Delta-like 1 (Dll1), Dll3, and Dll4] to a Notch receptor (Notch 1, 2, 3, and 4) on an adjacent signal-receiving cell. The triggered Notch receptor undergoes two sequential proteolytic cleavages to form a free Notch intracellular domain (NICD), the first cleavage in the extracellular region by ADAM10/17 (a disintegrin and metalloproteinase), and the second by γ-secretase activity of PS (preselin) within the transmembrane domain. The NICD translocates into the nucleus followed by interaction with a transcriptional repressor complex composed of CSL [C-promoter–binding factor (CDF-1) suppressor of Hairless/Lag-1; aka Recombining binding protein suppressor of hairless (RBP-J)] and other corepressors. The NICD–CSL interaction dissociates the repressor complex and recruits MAML (mastermind-like proteins) and coactivators such as p300 to release gene silencing and upregulate Notch target genes. Numb is an inhibitor of Notch signaling. In our proposed model for asymmetric cell division for latency, Notch signaling will be restricted in cells distal to the APC that contain Numb, maintaining Tcm and Tscm phenotype and HIV latency. Cells proximal to the APC will have decreased expression of Numb; thereby maintaining the capacity for Notch signaling, T-cell proliferation, NFκB activity, IFN-γ, and IL-2 production offering a favorable environment for HIV replication.

Canonical Notch signaling occurs after a cell presenting ligand [Jagged1, Jagged2, Delta-like 1 (Dll1), Dll3, and Dll4] binds to a Notch receptor (Notch 1, 2, 3, and 4) on an adjacent cell (*Fig.* 3). The triggered Notch receptor undergoes two sequential proteolytic cleavages to form a free Notch intracellular domain (NICD). The NICD translocates into the nucleus followed by interaction with a transcriptional repressor complex comprised of CSL [C-promoter–binding factor-1 (CDF-1) suppressor of Hairless/Lag-1, also called recombining binding protein suppressor of hairless (RBP-J)] and other corepressors. The NICD–CSL interaction dissociates the repressor complex and recruits MAML (mastermind-like proteins) and coactivators such as p300 to release gene silencing and upregulate Notch target genes 222, 223.

## Notch signaling in T-cell differentiation and quiescence

Preservation of long-lived quiescent T cells is dependent on inhibition of apoptosis and maintenance of cells in a non-dividing (G_0_) state. Notch signaling regulates both cell cycle and apoptosis. For example, target gene Hes1 (hairy and enhancer of split-1) downregulates Notch ligands and expression of G1 cyclins in mouse neural progenitors 224, and Mathieu *et al*. 225 identify that *Pdcd1* transcription of a negative regulator for T-cell activation, PD-1, is controlled through Notch signaling in activated CD8^+^ T cells. Several studies report that high levels of Notch signaling induce quiescence, whereas low levels promote proliferation and differentiation 226–228. Furthermore, a recent study reported that Notch protects expanded CD4^+^ T-cell clones from apoptosis and stimulates genes found in the metabolic pathway (i.e. carbohydrate, lipid, glucose metabolism, glucose transport, biosynthesis, and energy generation) 229.

Although Wnt and Notch are separate pathways, numerous reports suggest that these two pathways are interlinked in cell self-renewal, quiescence, and cell fate 220, 222. For instance, Wnt signaling upregulates Notch ligands Jag1 230, Dll4 231, and expression of the Notch2 receptor 232. In contrast, Wnt pathway component Dvl binds NICD to block Notch signaling 233. Additional interactions between NICD and Wnt proteins have also been shown to disrupt Notch activity. These include axin-NICD 234, axin and adenomatous polyposis coli-NICD 235, and GSK3β phosphorylation of NICD 236, 237. Preselin1 (PS1) is another protein with Wnt and Notch activity. PS1 is the γ-secretase involved in the intramembranous proteolysis of Notch within the signal-receiving cell to form the NICD; however, PS1 is also a negative regulator of β-catenin degradation in the canonical Wnt signaling pathway 238, 239.

The role of Notch in memory T-cell differentiation (Tn, Tcm, Tem, and Teff) has not yet been investigated. However, the following characteristics support the notion that Notch signaling may be linked to memory T-cell homeostasis: (i) Notch increases cell survival in T cells; (ii) high levels of Notch signaling induce cell quiescence; (iii) Wnt and Notch pathways regulate one another 220, 222; and (iv) regulation of Notch signaling is associated with asymmetric division, as discussed below 240–242. Combining these factors with studies in stem cells suggest that a versatile pathway such as Notch may also facilitate memory T-cell homeostasis and differentiation.

## Notch–Wnt signaling and inhibition of HIV replication

Not only are Notch and Wnt important in CD4^+^ T-memory cell development, proliferation, and survival, recent studies have shown that Notch and canonical Wnt signaling modulate HIV replication in various target cells. For instance, Tyagi and Karn 243 demonstrate that CBF-1 (C-promoter–binding factor-1, the mammalian representative of the CSL family) of the Notch signaling pathway reduces RNA polymerase II on the HIV promoter and recruits HDACs to the LTR in Jurkat cells, inhibiting HIV transcription. In other studies, β-catenin and TCF4 of the Wnt/β-catenin pathway are strong inhibitors of HIV replication in astrocytes and peripheral blood mononuclear cells 244–247. Of interest, IFN-γ, a type II interferon with antiviral activity that promotes proapoptotic response in infected cells, is found to be elevated in plasma, lymph nodes, and cerebrospinal fluid of HIV-infected individuals. IFN-γ upregulates expression of the Wnt/β-catenin pathway inhibitor Dkk1 through STAT3 signaling and enhances HIV replication 248. These data suggest that components of both the Notch and Wnt signaling pathways inhibit HIV replication, contributing to induction of HIV latency in CD4^+^ memory T cells, whereas components of immune response to infection (IFN-γ) antagonize Wnt signaling to increase HIV replication. It will be important to explore the mechanisms associated with Wnt/Notch signaling in HIV infection of CD4^+^ memory T cells and their relationship in HIV-infected populations (i.e. acute, chronic, elite controllers, and antiretroviral-treated immune responders or immune non-responders).

## Asymmetric cell division as an inducer of latency

Dividing stem cells must have capacity to produce progeny with differentiation potential and progeny that retain the parental, stem cell qualities. One mechanism through which this is possible is called asymmetric cell division. This is typically observed in stem cells that are capable of polarization such that the composition of one daughter cell is distinct from the other. In certain types of stem cells, this mechanism will generate one daughter cell that retains the original stem cell phenotype and another that has distinct effector cell function 249, 250.

Asymmetric division of this kind is perhaps most apparent in T cells because prolonged engagement of the TCR in an immune synapse creates a highly polarized cell for division. This polarization results in unequal distribution of proteins between the two daughter cells and generates one cell with effector function and one with memory function 240. Many molecules important to effector function and differentiation such as IFN-γR, PKC-ζ, T-bet, and CD25 segregate preferentially toward the immunological synapse and segregation of the proteasome is responsible, at least partially, for this effect 251, 252. The process of polarization of dividing cells persists even after the dissemination of the immunological synapse and is retained by the networks of the partitioning-defective protein 3 (Par3) and Scribble complexes 241.

Although the segregation of HIV-specific proteins toward the immunological synapse has not been characterized, it seems plausible given the polarization of viral particles toward the virological synapse. The possibility that HIV particles and proteins could accumulate at the interface with an APC suggests a very appealing mechanism by which a cell division would result a daughter cell with active HIV and one with latent HIV. Mathematical predictions have suggested that this concept of asymmetric division within HIV-infected cells may account for the kinetics of detectible virus in patients undergoing ART 253.

The basic model of asymmetric cell division in the maintenance of HIV latency is as follows. An HIV-infected CD4^+^ T cell engages its cognate antigen on an APC forming a stable immunological synapse. Along with cellular components that polarize toward the APC, HIV proteins and particles would also move toward the active TCR interface. After division, the daughter cell engaged with the APC would have the effector cell phenotype and would contain the active HIV. The daughter cell that is distal to the immunological synapse would maintain a memory phenotype, or possibly a stem cell memory phenotype, and would contain only the integrated HIV proviral DNA. In this case, the memory cell would lack the essential transcription machinery, such as Tat protein, to drive HIV virus production. If this model were accurate, it would predict that the latent HIV reservoir would be smaller in the effector cell population than in memory population, as the effector cells would contain mostly active virus and would be purged during ART. In fact, when the latent reservoir is quantified in antigen-experienced CD4^+^ T-cell subsets, the latent reservoir mostly persists in the less differentiated Tcm subset and is significantly decreased in Tem cells 17.

## Notch–Wnt signaling and asymmetric division in HIV latency

Unequal distribution of Numb, an inhibitor of notch signaling, is a classical indicator for asymmetric division in antigen-stimulated cells and essential in determining cell fate 240, 241. Numb is polarized to the distal side of a cell undergoing division, along with Par3 and atypical protein kinase C (aPKC), whereas Scribble and disks large family (DlgF) polarize to the proximal cell 241. In the proposed model for asymmetric cell division for HIV latency, Numb would be allocated to the cell distal to the APC, restricting Notch signaling in cells that maintain a memory or Tscm phenotype, and replicating HIV would be polarized to effector cells harboring ongoing Notch signaling (*Fig.* 3). Initial studies indicate that Notch signaling is required for CD4^+^ and CD8^+^ T-cell proliferation, NFκB activity and IFN-γ production 254, and will increase IL-2 receptor expression followed by production of IL-2 on CD4^+^ T cells 255. Inhibition of Notch signaling by Numb during asymmetric division would prevent activation and differentiation of distal daughter cells, contributing to the Tcm and Tscm phenotype.

Habib *et al*. 256 recently designed a single-cell embryonic stem cell *in vitro* model for Wnt-induced asymmetric division. Localized Wnt signal stimulates Wnt signaling ‘Wnt-on’ in the dividing cell proximal to Wnt3a ligand and maintains embryonic stem cell pluripotency, whereas the dividing cell distal to the Wnt3a ligand is in a ‘Wnt-off’ state and differentiates toward an epiblast stem cell (EpiSC) fate 256. To our knowledge, the role of Wnt3a in asymmetric cell division and its association with Numb and Notch signaling *in vivo* has not yet been investigated. Although these observations indicate a role for Notch and Wnt signaling to promote asymmetric division of memory CD4^+^ T cells and HIV latency, directed studies will help elucidate these novel hypotheses.

## Conclusion

The immunological synapse provides strong potential mechanisms for the establishment and maintenance of a latent HIV reservoir. The immunological synapse can dictate functionality of T cells through expression of a multitude of cytokines and cofactors. In the context of an HIV-infected T cell, this means that components of the immune synapse may be able to facilitate latency as well as active infection. In addition, the observation of asymmetric cell division in response to T-cell stimulation by the immune synapse provides a potential mechanism by which HIV infection can be maintained in a latent state, while at the same time producing progeny that actively express HIV virus. As the precise mechanisms of HIV latency establishment and maintenance become clearer, interventions that disrupt specific components of the immunological synapse may represent the final step in the process of HIV eradication.
